# To disclose or not to disclose: an ethnographic exploration of factors contributing to the (non) disclosure of Ghanaian women’s breast cancer diagnosis to social networks

**DOI:** 10.1186/s12905-023-02508-8

**Published:** 2023-07-10

**Authors:** Linda Serwaa Agyemang, Richard Wagland, Claire Foster, Chris McLean, Deborah Fenlon

**Affiliations:** 1 Department of Nursing Science, Faculty of Health and Social Science, Bournemouth University, Bournemouth, UK; 2 Centre for Psychosocial Research in Cancer, Health Sciences, Faculty of Environmental and Life Sciences, University of Southampton, Southampton, UK; 3 Health Sciences, Faculty of Environmental and Life Sciences, University of Southampton, Southampton, UK; 4 Department of Nursing, College of Human and Health Science, University of Swansea, Swansea, UK

**Keywords:** Breast cancer, Ghana, Ethnography, Disclosure, Stigma, Spiritual beliefs, Safe space, Informational support, Financial support, Social network

## Abstract

**Background:**

Although there may be theoretical support linking positive health outcomes with cancer disclosure to social networks, women from contexts such as Ghana where cancer is not openly talked about may have concerns around breast cancer disclosure. Women may not be able to share their experiences about their diagnosis, which may prevent them from receiving support. This study aimed to obtain the views of Ghanaian women diagnosed with breast cancer about factors contributing to (non) disclosure.

**Methods:**

This study is based on secondary findings from an ethnographic study that employed participant observation and semi-structured face to face interviews. The study was conducted at a breast clinic in a Teaching Hospital in southern Ghana. 16 women diagnosed with breast cancer (up to stage 3); five relatives nominated by these women and ten healthcare professionals (HCPs) participated in the study. Factors contributing to breast cancer (non) disclosure were explored. Data were analysed using a thematic approach.

**Results:**

The analysis indicated that most of the women and family members were very reticent about breast cancer disclosure and were secretive with distant relatives and wider social networks. Whilst remaining silent about their cancer diagnosis helped women protect their identities, prevented spiritual attack, and bad advice, the need for emotional and financial support for cancer treatment triggered disclosure to close family, friends, and pastors. Some women were discouraged from persevering with conventional treatment following disclosure to their close relatives.

**Conclusions:**

Breast cancer stigma and fears around disclosure hindered women from disclosing to individuals in their social networks. Women disclosed to their close relatives for support, but this was not always safe. Health care professionals are well placed to explore women’s concerns and facilitate disclosure within safe spaces to enhance engagement with breast cancer care services.

**Supplementary Information:**

The online version contains supplementary material available at 10.1186/s12905-023-02508-8.

## Background

Receiving a cancer diagnosis is distressing and can have many emotional, physical and psychological consequences [1, 2]. With regards to breast cancer, this distress may be profound for some women because the disease is seen not only as a threat to life but also to femininity and sense of womanhood [3, 4]. Across many cultures, Ghana is not an exception, women are socialised to perceive their personality to be reflected in their external appearance and that their breasts are not exclusively their own but exist for the evaluation and gratification of others [3]. Consequently, the thought of losing a breast may be devastating. The benefits of disclosure, i.e. openly sharing one’s cancer diagnosis and concerns about the disease has been highlighted in the literature [2].

Some studies have stressed that disclosure may help an individual to better reorganise their thoughts and make sense of their cancer experience [1, 2, 5]. In a study conducted by Edward and Clark [6] in Australia, the authors emphasised that patients who have good communication with their family and loved ones have less psychological distress than those who have not. In a qualitative study by Anarado et al. [7], the authors found that among Nigerian women with breast cancer, sharing concerns with survivors about their diagnosis facilitated the receipt of information that encouraged them to proceed with conventional treatment. Ntekim et al. [8] and Mc Ewan et al. [9] reported that patients who disclosed their diagnosis to family and friends in Nigeria and Egypt respectively, received financial assistance which enabled them to access treatments.

While the above studies [5–9] demonstrated that breast cancer disclosure may lead to improved health outcomes, they also emphasize that those disclosures occurred within safe spaces. Indeed, some authors have stressed that disclosures within certain parameters might be problematic as there is potential for a stressful and hostile environment should a woman’s breast cancer diagnosis become public [10]. In Ghana, negative responses such as stigmatisation and exclusion may deter women from sharing their breast cancer diagnosis to members within their community [11, 12]. It is argued that traditional notions around the full woman (a woman with two breasts) and socio-cultural beliefs about gender roles have implications for discourses around breast cancer in Ghanaian communities [12]. In Ethiopia, De Ver Dye et al. [13] found that participants with breast cancer did not have active discussions around cancer in their communities. Participants described how discussion of breast cancer seemed socially forbidden and consequently, women hid their diagnosis which in turn delayed engagement with cancer care services [13].

The above issues highlight the complexities around disclosure of breast cancer diagnosis to social networks and particularly amongst women in West Africa. However, little is known about women in Ghana. Agyemang et al. [14] identified a pattern of secrecy around breast cancer diagnosis. This paper reports on the secondary findings of Agyemang et al. [14] regarding the views of women about (non) disclosure of their cancer diagnosis to members of their social network (including close and distant relations, friends, colleagues and peers), and the factors influencing such decisions.

## Methods

This article reports secondary findings from an ethnography conducted at a breast clinic in a Teaching Hospital in southern Ghana [14]. The study involved participant observation and semi-structured interviews of women with breast cancer, nominated relatives and healthcare professionals (HCPS) about the factors influencing women’s treatment decision making [14].

During the conduct of the ethnography, factors such as hierarchical relationship and structural constraints ensured hidden information and secrecy around breast cancer in the clinic environment [14]. This was explored further using semi-structured interviews to understand the factors contributing to breast cancer (non) disclosure outside the clinic environment.

### Setting

The hospital, where the breast clinic is located, is situated in a metropolitan city and serves as a cancer referral centre for the northern and middle parts of the country. The hospital is accessed by people with diverse socio-economic, education, religious and ethnic backgrounds. In addition, breast cancer is the most common cancer presented there [15].

### Sampling

Purposive sampling was employed to enrol participants into the study. The criteria were to include women 18 years and over, diagnosed with breast cancer (up to stage 3) and who could speak Twi (a local language commonly spoken in Ghana) or English [14]. The study also included relatives because they often accompanied women to the clinic. The relatives were included only if women gave consent and nominated them. HCPs (nurses and doctors) were included in the study because they were involved in providing breast cancer diagnosis and treatment recommendations to women.

### Data collection

Breast cancer diagnosis and treatment recommendations occurred at the breast clinic. Fieldwork involved participant observation of the interactions between women, nominated relatives and HCPs at the breast clinic. This was conducted by LSA (a female registered adult nurse who is also a native Twi speaker, unknown to participants prior to the study) between July 2017 and November 2017. Observations were followed by semi-structured face-to-face interviews, conducted by LSA using an interview guide (Additional File 1), which was pilot tested on one nurse and one patient participant. The guide was informed by a previous literature review [16] and by emergent issues. The interview guide was written in English, but translated into the Twi Language during interviews, unless participants opted to speak English. Interviews were conducted in the hospital, participants’ homes or workplaces [17, 18] according to participants’ preference. Interviews were audio recorded and field notes were compiled on observations, context, personal reflections and self-evaluations. Audio recordings were transcribed verbatim in ‘Twi’ before translation by LSA into English. A validation check from a bilingual local expert was sought for translation accuracy. Interviews were conducted from between four to twelve weeks post diagnosis and lasted between 45 and 60 min. To maintain anonymity, each participant was assigned a unique pseudonym [16].

### Data analysis

Analysis of the data was led by LSA using thematic analysis [16], assisted with computer software NVivo 12 [19]. The analysis was iterative which involved making sense of emerging issues (e.g., secrecy) in the data to guide the next stage of data collection. This process continued until data saturation was achieved. During the process, initial codes were assigned to reflect the predominant issues present in the data [16]. Similar codes were conceptualised into larger categories, which allowed broad patterns to emerge and developed into themes. Two of the co-authors (RW and CF) assessed a portion of the transcripts with the generated codes to ensure they reflected the data.

### Ethics

Ethical approval was obtained from the University of Southampton (Ethics ID: 26,346) and Kwame Nkrumah University of Science and Technology (Reference: CHRPE/AP/393/17). The researcher (LSA) accessed the breast clinic through a gatekeeper who introduced her to the breast clinic unit head who in turn introduced her to the rest of the staff at the breast clinic including the breast clinic nurse in-charge, doctors, staff nurses and the non-clinical staff on the unit. Meeting with the staff on the unit allowed her to gain support from them for the study. Nurses at the clinic informed potential participants about the study. Patient participants who expressed interest in the study were asked to contact the researcher face-to-face after they had received their routine care for the day. All potential participants were provided with plain language information sheets outlining the aim and purpose of the study. For potential participants without formal education, information was read to them in the local Twi Language. They were then asked to give the information sheets to relatives and to discuss with them before deciding to participate. All potential participants were given at least 24 h to decide whether or not to take part and written consent was obtained before participation. Participants were informed of their right to refuse participation [20].

### Credibility

Trustworthiness and rigour of this study was ensured by following the strategies proposed by Lincoln and Guba [21]. Participant observation and in-depth interviews ensured familiarisation with participants during breast cancer diagnosis and treatment recommendations. There was prolonged engagement in the field which ensured data saturation and adequate data to support the analysis. Another strategy employed was involving more than one group of participants (i.e., patients, families, HCPs) to facilitate a broader understanding of the phenomenon under investigation. Also, feedback from co-authors was regularly obtained to enhance different views and ideas for additional exploration, and appropriate interpretation of data [22]. This study was reported according to the Consolidated Criteria for Reporting Qualitative studies [COREQ] [23], which allowed for accurate reporting and discussion of the similarities between this study’s findings and those of other studies.

## Findings

Between July 2017 and November 2017, approximately 89 h of participant observation occurred at the breast clinic over 49 days. 31 participants (16 women diagnosed with curative breast cancer and five nominated relatives; 10 HCPs) were involved in observations and 29 were interviewed. Two HCPs were not available for interviews due to work demands. Table 1 provides a summary of participants’ characteristics.

Four main themes around breast cancer disclosure emerged from the data: (i) social stigma, (ii) fear of spiritual attack, (iii) preventing bad advice and (iv) trust in receiving help.

**Table 1:** Participant characteristics

Characteristics of patient participants	Category of characteristics	Number of participants
Age (years)	20–30	1
	31–40	3
	41–50	4
	51–60	3
	61–70	2
	71–80	2
	81–90	1
Education	No formal education	3
	Primary or elementary	9
	Secondary	1
	Tertiary	3
Monthly income of patient participants (US Dollars)	None	2
	Irregular	9
	85–170	4
	171–499	0
	500–850	1
Breast cancer stage	Stage 2	7
	Stage 3	9
Recommended treatment	Breast conservation, adjuvant	1
	Mastectomy	1
	Mastectomy, adjuvant	3
	Neoadjuvant, mastectomy, adjuvant	11
**Characteristics of nominated relatives**	**Category of characteristics**	**Number of participants**
Age	Median 32 years; Range 23 years	5
Formal Education	Primary or elementary	3
	Secondary	1
	Tertiary	1
Monthly income (US Dollars)	Irregular	3
	50–99	1
	100–199	0
	200–250	1
**Characteristics of HCP participants**	**Category of characteristics**	**Number of participants**
Professional/clinic role	Consultant general surgeon	2
	Resident	3
	Adult general nurse	4
	Nurse/Midwife	1
Years in service	10 or more	3
	> 5 < 10	3
	Up to 5	4

### Social stigma

Most of the patients and relatives did not want to disclose their cancer diagnosis to individuals within their social network due to stigma. They stressed that cancer has a poor prognosis driven by the high mortality rate associated with the disease. Two participants (one patient and one relative) expressed their views that disclosing the diagnosis means people in their community would talk about them and would not expect them to survive.

*I do not want others to know because if you tell people, it will go public and everyone will say ei! [paused for a second and raised her eyebrows] this woman is suffering from cancer, she will not live long (Maame Mary, 68 years).*

This perceived social reaction was accepted and internalised by two other women as true.

*“…you spend all this money on treatment and die anyway, so, I will hang on, when I die, I die” (Akos, 38 years). Sister Akos turned her gaze away from me as she spoke. Field note.*

Many of the participants mentioned their reason for non-disclosure as preventing shame and exclusion. One woman described how news of her diagnosis would mean people would not want to associate with her and that she would be treated as ‘filthy’.

*if someone hears it, they will ‘declare’ you that you are unworthy, you are not part of them, nobody wants to get close to you because you have this cancer, the person will not be happy to come close to you and so you will be treated filthy (Foriwaa, 48 years).*

Stigma was also associated with a loss of identity and one woman described that she did not want other people in the community to know about her breast cancer diagnosis as she would be ridiculed because ‘one of her breasts has been cut’.

*if I tell people I have cancer, they will go about telling other people, spreading the news everywhere that this woman is suffering from cancer… the doctors have cut my breast, people will spread the news that they have cut my breast (Maame Tawia, 52 years).*

The perceived stigma may not only be directed at the individual with cancer but could potentially be directed towards the family. Family members were concerned about disclosure because they feared their family would also be stigmatised.

*I have told my mother not to tell anyone that she has cancer,…we want the diagnosis to stay within the family but not for outsiders to know about our family issues…every family has a ‘Mensah’ (and we don’t want it to spread outside …and people talking about it (“Mensah” means a bad nut or social deviant in a local Ghanaian context) (Maame Mary’s daughter, 30 years).*

The perceived social stigma was not only enacted from verbal disclosures but also through the possession of breast cancer information leaflets. Nurses at the breast clinic recounted women refused information leaflets or left them in the clinic because they feared the leaflets would link them to the disease, which could cause them to be stigmatised. This was recorded in field notes.

*During the time I have been in the breast clinic, I did not observe patients being given any supplementary breast cancer information leaflet. On one afternoon, during casual conversation with the nurses at the clinic about whether they provided supplementary information materials, the charge nurse went into her office and brought out several dozens of information leaflets on breast cancer. The leaflets were of the same kind written in English Language. She gave one to me and said that they stopped sharing leaflets to the women as the women either refused them or left them in the clinic. I asked if she knew why and she mentioned in a rather loud voice, “they won’t take it because of stigma” (Field note).*

The perception of the nurses was that breast cancer information leaflets could become an indirect means of disclosing women’s cancer diagnosis to the public and could be a basis for stigmatising behaviours towards the women.

On the whole, due to the fear of stigma, there is unwillingness to talk about the disease to other people in the community. The lack of open discussions about the disease means people are not able to share their experiences about it.

### Fear of spiritual attack

Fear of disclosure was also motivated by a widespread belief that some people possess evil supernatural forces, and if they become aware of a person’s diagnosis, they will employ evil means spiritually to kill them in the spiritual realm. Due to this widespread belief, there is general unwillingness to talk about cancer. Many of the patients interviewed feared that when people who possess evil spirits hear of a person’s diagnosis, they can use their influence with supernatural forces to prevent a cure. As a result, the patient may have all the treatment but will never get cured, rather, it would lead to impoverishment as the patient spends all her money on treatment. For these reasons, many of the patients did not want to disclose their diagnosis to anyone other than trusted friends and relatives and often used metaphors such as, *‘I do not want to sell my story’*. For many of the patients and relatives, if you ‘sell your story’, the ‘buyer’ can choose to do anything they want with it, including ‘killing you’ or ‘preventing cure’.

*the reason why I don’t tell someone is because it is not every issue that we tell people…some people when you tell them about your issue, the person may have evil spirit… they can do evil incantations in the spirit to block your chance for a successful recovery…that is why we don’t sell our story (Maame Saa, 72 years).*

Such superstitious beliefs limit open discussions about the disease in the community.

### Preventing bad advice

Women often avoided disclosure because they feared they would likely receive bad advice from their peers with regards choice of treatment. There is a strong belief amongst many Ghanaian people in the efficacy of spiritual and herbal healing. Disclosing one’s diagnosis gives other people the opportunity to persuade them to use different kinds of healing modalities rather than mainstream biomedical treatments. One relative described concern about his mother sharing her diagnosis with her pastor as he feared the pastor would encourage spiritual healing rather than conventional treatment. Similarly, a sibling advised his sister against disclosing to anyone to prevent her from being advised to use herbal treatment.

*I think in this country when you are sick people will keep saying go and do herbal treatment and all that, that is why I discourage my sister from talking to other people about it (Lydia’s brother, 52 years).*

Concerns about patients receiving bad advice from others was not only limited to patients and relatives but were shared with HCPs. This was documented in the field notes when Dr E was interacting with Sister Lydia.

*Following diagnosis, Dr E cautioned Sister Lydia not to disclose the diagnosis to her mother because she would wrongfully advise her to choose herbal healing. “…when you go home, … do not tell your mother about your condition, because your mother will tell you to go for herbal treatment and that will not help you*” (Field notes).

### Trust in receiving help

Although generally, women and their relatives fear disclosure of breast cancer diagnosis, many of the women who participated in the study mentioned they had disclosed their diagnosis to significant others (a relative, friend or pastor) whom they trusted could provide help. The study revealed three main factors that contributed to disclosure, and this included receiving, financial, emotional and informational support.

#### Financial support

Breast cancer treatment is cost intensive in Ghana regardless of whether the woman is covered by the National Health Insurance Scheme. Hence, some women disclosed their diagnosis to their significant others to mobilise funds to support treatment.

*…my mother wants the good of me and my children and she even assists me financially… it is only my mother I have discussed it with… (Akos, 38 years)*.

*I have told my elder sister and she is helping, even the labs I did, she was the one who paid for them (Adwoa, 32 years)*.

Other women have relatives abroad with greater resources who loaned them monies when they become aware of the diagnosis.

*…I told my nephew, he lives abroad, he also helped me and supported me with money to come to hospital (Maame Tawia, 52 years)*.

#### Emotional support

Breast cancer is a life-threatening disease and as such diagnosis can evoke fears and worries about dying and/ or treatment side effects. Some women therefore disclosed their diagnosis to receive emotional support.

*When I went home too, I informed my husband about it…I was very sad and I started crying and he said I should stop crying and that because of God, nothing will happen to me (Auntie Twumwaa, 56 years)*.

Other women disclosed to their Pastors who support them with prayers.

*I informed my pastor…he has been praying with me* (Foriwaa, 48 years).

Other patients talked to survivors who had completed some phases of treatment. Survivors gave women hope for cure and encouraged them to persevere with the recommended treatment. For example, Natasha was initially concerned about the side effects of chemotherapy, but after sharing her experience with a survivor, she developed a positive outlook about it.

*…when I was told the diagnosis, I was scared of the chemo because of the side effect…it can affect my hair….so the chemo puts me down…but when I talked about it with the survivor and seeing that she had it but she is fine, …I think it is a good treatment… (Natasha, 28 years)*.

#### Informational support

Some women were concerned they had very little knowledge about the disease, so they disclosed their diagnosis to others who they believed could provide them with information.

*…when I heard the word cancer, I thought I was going to die…I wanted more information later on…so I talked to my employer’s doctor who said everything was going to be fine that I can do the treatment…he even said if I wanted to give birth again, I can do it (Adwoa, 32 years).*

Overall, women tend to only disclose their cancer diagnosis to people they trust can help them. The attitudes of these individuals towards breast cancer treatment will therefore be very influential in women’s treatment decisions.

*my mother wants the good of me and my children… she even assists me financially.…So when I came to the breast clinic, I told my mother what the doctors told me… My mother said that if it is only the injections, (chemotherapy), …I will get and be well, then she will look for money for me to get the treatment… but if after getting all these injections, they will remove the breast as well, my mother said it is not necessary for me to go through that treatment… (Akos, 38 years).*

## Discussion

This study set out to explore factors contributing to breast cancer disclosure and found that openly sharing one’s diagnosis (or not) is a deliberate, thoughtful and calculated decision for Ghanaian women with breast cancer. A general hesitancy was consistent amongst the participants, and this highlights the barriers associated with breast cancer disclosure in Ghanaian society. Previous studies conducted elsewhere in Ethiopia and Kenya identified stigmatisation as a common concern among breast cancer patients [13, 24] and this has been shown to be linked with non-disclosure [24]. The findings of the current study appear similar to those conducted in Uganda where the belief in the certainty of cancer death resulted in self and public stigmatisation towards women diagnosed with breast cancer [10]. Breast cancer is the most common cause of cancer death among women worldwide [25] so the association of breast cancer with death is not particular to women living in developing countries. However, importantly in developing countries, including Ghana, breast cancer is often diagnosed at an advanced stage with poorer prognosis [26]. Consequently, this reinforces local assumptions that cancer means death and a basis for stigmatising behaviours towards diagnosed women. Stigmatisation is also associated with changes in body image that resulted from treatment [10, 13, 27]. The loss of a breast and the consequent shame of being ridiculed and labelled as ‘unworthy’ can lead to internalised stigma [28], which in turn can lead to the erosion of one’s sense of self-worth and negative feelings about oneself [29]. A systematic review by Taft and Keefer [30] demonstrated that illness stigmatisation correlates with poor health outcomes from limited access to medical care, treatment nonadherence and psychological distress. The attribution of negative attitudes and stereotypes about breast cancer and its treatment also reflects society’s construction of the acceptable image of a woman as one with two breasts [12]. This widely held idealised image of the female may compel women with breast cancer to hide themselves from spheres of society because of stigma [10, 12].

The Ghana Ministry of Health should promote breast cancer educational messages in public places to increase awareness, challenge stigma and dispel misconceptions about the disease [10, 31]. Furthermore, the ministry should develop policies [10, 31] with targeted educational programmes to focus on the existence of breast cancer survivors within the community and the importance of early detection and treatment on survival [11].

Another finding was that women were reluctant to disclose to social networks other than trusted family and friends due to concerns that they could be persuaded to exclusively use non-conventional treatment. While this may be beneficial in keeping women safe and protect them from non-engagement with cancer care services, the secrecy around breast cancer could contribute to a continued lack of awareness that cancer survivors exist within the communities. This reinforces local misconceptions about the disease. At the practice level, HCPs are positioned to support women to identify safe spaces such as breast cancer survivorship groups for disclosure. Policies aimed at empowering women to improve their knowledge about breast cancer are encouraged.

The study also found that women shared their breast cancer diagnosis at a time when they are at their most vulnerable. Diagnosis is shared with the aim of receiving some kind of help, which was mostly financial, but also included emotional and informational help. Cancer disclosure and the consequent financial and informational support women received from their trusted relatives, friends and pastors, dictated their course of action. While most of the women in the current study were encouraged to opt for conventional treatment, a few were advised to use either herbal and/or spiritual healing. Thus, a woman’s disclosure to an otherwise trusted person may not always be safe and may even be counterproductive, leading to delayed engagement with cancer care services. This finding has been reported in other studies from the African continent [32, 33] and has been linked with delayed breast cancer presentations. Interestingly, women in this study rarely mentioned that they had consulted HCPs at the breast clinic when they needed further information, and this possibly reflects barriers in patient-provider communication [14]. These findings have strong implications for HCPs to be aware of the vulnerabilities of women and are encouraged to prioritise those who are more likely to be misinformed and provide appropriate informational support. HCPs are well placed to explore women’s emotional needs and as mentioned above, help women identify safe spaces to share these concerns and receive the needed support. There are also implications for policymakers to address financial barriers in cancer treatment [34].

This study has several strengths including immersion in the natural setting of participants; sampling participants from a hospital widely accessed by a heterogeneous group of people; and involving more than one group of participants. These approaches facilitated a broader understanding of the different perspectives impacting breast cancer disclosure [35]. The status of the primary author as both an insider and outsider was a strength for this study. Her insider status (Native Ghanaian nurse) allowed an understanding of the common local language and culture, which facilitated a trusting relationship with participants and allowed interaction with participants without any hindrance. On the other hand, her status as an outsider (because of exposure to 6 years of UK higher education and culture), facilitated insights into issues that would otherwise have gone unnoticed.

A potential limitation of the study was that the participants were from a single tertiary hospital and the majority (88%) had opted for conventional treatment, therefore, the views of participants who did not report to this facility are not known. Another limitation was that although participants’ educational background and marital status may have impacted upon their beliefs and strategies, these were not explored in-depth. A further limitation was the need to translate interview data from Twi to English as it raises the question as to whether translated versions retained the values, assumptions and feelings that were present in the original spoken language version [36]. This potential limitation was unavoidable but was offset by both the lead author’s insider status and the dual role of researcher/translator, and by employing validation checks from a local bilingual language expert [37].

## Conclusion

This study has identified that stigma and fears around breast cancer disclosure prevented women from disclosing their diagnosis to distant relatives and wider social networks and they only disclosed to their trusted friends, family, peers, and pastors. However, this trusted support may not always be safe and could lead to delayed cancer care. HCPs can explore women’s concerns and help identify supportive safe spaces like breast cancer survivorship groups and advocacy groups. Psychologists and counsellors could also work with staff in the breast clinic to provide counselling and emotional support to women with fears and worries.

## Electronic supplementary material

Below is the link to the electronic supplementary material.

Additional File 1: Interview guide topic

## Data Availability

The dataset used during the current study are available from the corresponding author upon reasonable request.
