# Supplementary material for: To disclose or not to disclose: an ethnographic exploration of factors contributing to the (non) disclosure of Ghanaian women’s breast cancer diagnosis to social networks
Source: BMC Womens Health. 2023 Jul 10;23:366. doi: 10.1186/s12905-023-02508-8 (PMC10334502; doi:10.1186/s12905-023-02508-8)
Supplement: Supplementary file 1 — Additional File 1: Interview guide topic [file 12905_2023_2508_MOESM1_ESM.docx]

Supplementary File 1: Interview guide topic

| Demographic information |  |
| --- | --- |
| Age | Income |
| Educational status |  |
| Patient participant Prompt | 1. Can you describe your thoughts about what the doctor and the nurse talked to you regarding breast cancer and treatment? Prompts: 2. What are your views about disclosing the breast cancer diagnosis to members of your social network? 3. Show series of pictorial drawing of social relations to woman and let her point to those she has (or has not) shared her concerns with and why? |
| Nominated Relative Prompt | 1. Can you share with me your views about breast cancer and treatment**?** 2. Can you tell me your views about breast cancer disclosure? Prompt: How do your views and concerns about breast cancer influence your support to the woman i.e. financial support, emotional support?) |
